# FJU-C4, a New 2-Pyridone Compound, Attenuates Lipopolysaccharide-Induced Systemic Inflammation via p38MAPK and NF-κB in Mice

**DOI:** 10.1371/journal.pone.0082877

**Published:** 2013-12-23

**Authors:** Jung-Sen Liu, Fang Jung, Shih-Hsing Yang, Shang-Shing P. Chou, Jhih-Liang Huang, Chang-Lin Lu, Guan-Lin Huang, Pan-Chyr Yang, Jau-Chen Lin, Guey-Mei Jow

**Affiliations:** 1 Department of Respiratory Therapy, Fu-Jen Catholic University, New Taipei, Taiwan; 2 Department of Chemistry, Fu-Jen Catholic University, New Taipei, Taiwan; 3 School of Medicine, National Taiwan University, Taipei, Taiwan; 4 School of Medicine, Fu-Jen Catholic University, New Taipei, Taiwan; Universidade Federal do Rio de Janeiro, Brazil

## Abstract

Despite advances in antibiotic therapy and intensive care, the mortality caused by systemic inflammatory response syndrome and severe sepsis remains high. The use of anti-inflammatory agents to attenuate inflammatory response during acute systemic inflammatory reactions may improve survival rates. Here we show that a newly synthesized 2-pyridone compound (FJU-C4) can suppress the expression of late inflammatory mediators such as iNOS and COX-2 in murine macrophages. The pro-inflammatory cytokines, including TNFα, IL-1β, and IL-6, were dose-dependently suppressed by FJU-C4 both in mRNA and protein levels. In addition, the expression of TNFα was inhibited from as early as 2 hours after exposure to LPS stimulation. The production of mature pro-inflammatory cytokines was also suppressed by pretreatment with FJU-C4 in either cell culture medium or mice serum when stimulated by LPS. FJU-C4 prolongs mouse survival and prevents mouse death from LPS-induced systemic inflammation when the dose of FJU-C4 is over 5 mg/kg. The activities of ERK, JNK, and p38MAPK were induced by LPS stimulation on murine macrophage cell line, but only p38MAPK signaling was dramatically suppressed by pretreatment with the FJU-C4 compound in a dose-dependent manner. NF-κB activation also was suppressed by FJU-C4 compound. These findings suggest that the FJU-C4 compound may act as a promising therapeutic agent against inflammatory diseases by inhibiting the p38MAPK and NF-κB signaling pathway.

## Introduction

Excessive inflammatory response induced by infection, chemicals, toxins, and cytokines may cause human diseases such as endotoxemia and systemic inflammatory response syndrome (SIRS) [1]. Despite advances in antibiotic therapy and intensive care, the mortality caused by SIRS and severe sepsis remains high [2], [3]. Macrophages play a critical role in human immune response to bacterial infection. Pro-inflammatory cytokines, such as tumor necrosis factor-alpha (TNFα) [4], interleukin-1beta (IL-1β) [5], and interleukin-6 (IL-6) [6], stimulated by the endotoxin lipopolysaccharide (LPS), extend inflammatory responses by activating other mediators, such as prostaglandins (PGEs) and nitric oxide (NO), which further promote inflammation, tissue damage, and death. Previous studies have shown that the use of anti-inflammatory agents to attenuate inflammatory response during acute lung injury can reduce mortality and prolong patient survival [7], [8]. The clinical use of immunosuppressive drugs with diverse anti-inflammatory mechanisms, such as cyclosporine A, rapamycin, and FK-506 have been shown to inhibit inflammatory response in macrophages; however, such drugs are unable to completely inhibit the expression and activity of inducible nitric oxide synthase (iNOS) and cyclooxygenase-2 (COX-2) [9]. Developing effective therapeutics that target inflammatory mediators is difficult because of the early release of pro-inflammatory cytokines (TNFα and IL-1β) in the development of systemic inflammatory response. Nevertheless, highly potent anti-inflammatory compounds for the treatment of human diseases with excessive inflammatory response such as sepsis and acute lung injury must still be developed.

Indolizidine and quinolizidine structures contribute promising anti-inflammatory and anti-cancer activities for clinical use, and they are worthy of further development [10]. The biological function and underlying mechanisms of these compounds against inflammation remain unknown. We synthesized a series of quinolizinone and pyridone derivatives based on the previous methods [11] and evaluated their biological function in anti-inflammatory responses. This study investigated the underlying effects and mechanisms of these newly synthesized compounds in anti-inflammatory responses stimulated by lipopolysaccharide in a murine macrophage cell line and animal model.

## Materials and Methods

### Cell culture

Raw 264.7 murine macrophage cells were purchased from Bioresource Collection and Research Center (Hsinchu, Taiwan). The macrophage cells were cultured in Dulbecco's modified Eagle serum (DMEM; Hyclone, Logan, UT, USA) supplemented with 10% fetal bovine serum (FBS, Hyclone), MEM non-essential amino acid (Hyclone), 100 mM sodium pyruvate (Hyclone), and antibiotics (Hyclone), and incubated at 37°C under an atmosphere of 5% CO2 and 95% air.

### Chemicals

**Figure 1:**
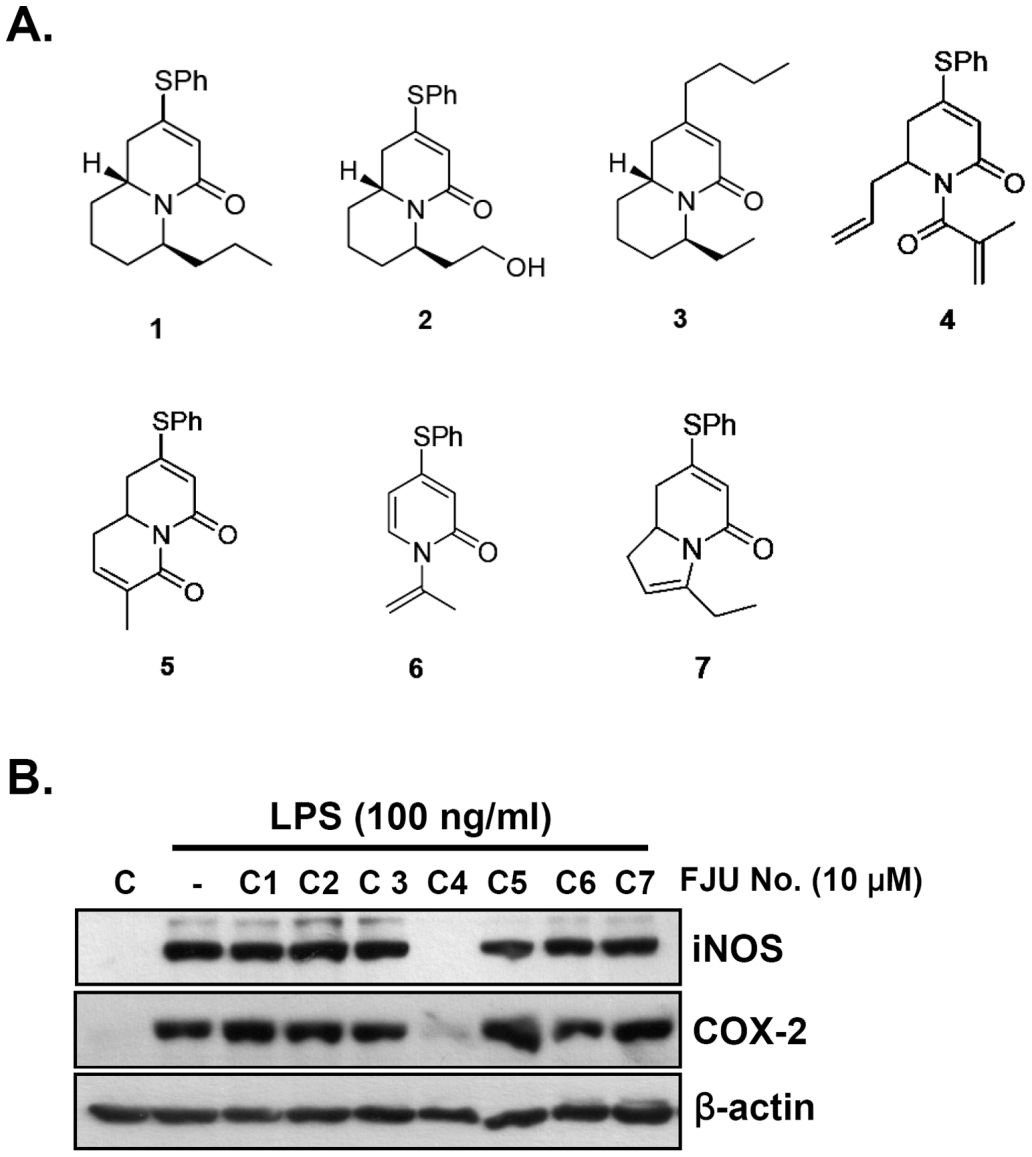
Structure and inhibitory effect of FJU-series compounds on iNOS and COX2 expression in Raw264.7 murine macrophages. (A) Chemical structure of synthesized quinolizinone and 2-pyridone derivatives. (B) Raw264.7 macrophage cells were pretreated with 10 μM of various derivative compounds for 30 min and then stimulated by LPS (100 ng/ml) for 24 h. The treated cells were analyzed by western blotting. Ph: phenyl (C6H5).

A series of quinolizinone and pyridone relative compounds (FJU-C1 to C7) were synthesized, as shown in Fig. 1A [11], [12], [13], [14], [15] and their name of these chemical compounds were listed on Table 1. Lipopolysaccharides (LPS, Escherichia Coli 0111:B4) were purchased from Sigma-Aldrich (Saint Louis, MO, USA).

**Table 1:** List of new synthesized derivative compounds.

No.	Name of chemical compounds
1	trans-6-Propyl-2-(phenylthio)-1,6,7,8,9,9a-hexahydro-4-quinolizinone
2	trans-6-(2-Hydroxyethyl)-2-(phenylthio)-1,6,7,8,9,9a-hexahydro-4-quinolizinone
3	trans-2-Butyl-6-ethyl-1,6,7,8,9,9a-hexahydro-4-quinolizinone
4	6-Allyl-1-(2-methylacryloyl)-4-(phenylthio)-5,6-dihydro-2-pyridone
5	7-Methyl-2-(phenylthio)-1,6,9,9a-tetrahydro-4,6-quinolizinedione
6	1-(1-Methylethenyl)-4-(Phenylthio)-2-pyridone
7	3-Ethyl-7-(phenylthio)-1,5,8,8a-tetrahydro-2-indolizinone

### RNA isolation and Reverse Transcription-Polymerase Chain Reaction

The cultured cells were washed with cold TBS (Amresco, Solon, Ohio, USA) buffer twice and then harvested for RNA isolation using Trizol reagent (Invitrogen, Carlsbad, CA, USA) following the manufacturer's recommended procedure. Total RNA (1 μg) was reverse-transcribed using random primers and an MMLV reverse transcriptase kit (Epicentre Biotechnologies, Madison, Wisconsin, USA), following the manufacturer's recommended procedure. The reverse transcription mixture (2 μl) was assayed to detect the mRNA level of the PCR primer sets (Table 2). The results were separated by electrophoresis on a 1% agarose gel and visualized by ethidium bromide staining. To quantify the expression of genes, real-time quantitative RT-PCR was performed, following our previously described method [16]. The specific primers were designed by qPrimerDepot (http://primerdepot.nci.nih.gov/) (Table 2). All reactions were performed in 20 μl volumes containing 10 μl of Realtime PCR Master Mix (SYBR Green) (Toyobo, Osaka, Japan). The expression of β-actin was used as an internal control for RNA quantity.

**Table 2:** Designed primers for RT-PCR.

Gene	Forward Primer (5′)	Reverse Primer (3′)		Size (bp)
**Primers for PCR**				
iNOS	CCCTTCCGAAGTTTCTGGCAGCAGC	GGCTGTCAGAGCCTCGTGGCTTTGG		497
COX2	GATGTTTGCATTCTTTGCCC	GGCGCAGTTTATGTTGTCTG		149
TNFα	TTCTGTCTACTGAACTTCGGGGTGATCGGTCC	GTATGAGATAGCAAATCGGCTGACGGTGTGGG		354
IL-1β	ATGGCAACTGTTCCTGAACTCAACT	CAGGACAGGTATAGATTCTTTCCTTT		563
IL-6	ATGAAGTTCCTCTCTGCAAGAGACT	CACTAGGTTTGCCGAGTAGATCTC		638
**Primers for Real-time PCR**				
iNOS	GAAGAAAACCCCTTGTGCTG		GTCGATGTCACATGCAGCTT	138
COX2	GATGTTTGCATTCTTTGCCC		GGCGCAGTTTATGTTGTCTG	149
TNFα	CGCTCTTCTGTCTACTGAACTT		ATGAGATAGCAAATCGGCTGAC	357
IL-1β	CGCAGCAGCACATCAACAAGAGC		TGTCCTCATCCTGGAAGGTCCACG	111
IL-6	CACAAGTCCGGAGAGGAGAC		CAGAATTGCCATTGCACAAC	141
β-actin	GATTACTGCTCTGGCTCCTAGC		GACTCATCGTACTCCTGCTTGC	147

### Enzyme-linked immunosorbent assay (ELISA)

TNFα, IL-1β, and IL-6 secretion were measured by ELISA kit (eBioscience, San Diego, CA, USA), following the manufacturer's recommended procedure. In the assay, 96-well plates were coated with monoclonal antibody with specificity for TNFα, IL-1β, and IL-6. The coated plates were washed 5 times with wash buffer between steps. The substrate solution was added to each well, and the plate was incubated at room temperature for approximately 30 min. The developed color was measured by a microplate reader (BioTek Instruments, Inc., Winooski, VT, USA). The concentration of TNFα, IL-1β, and IL-6 was determined by the standard curve.

### Western blot analysis

Total cellular protein from cells was extracted by PRO-PREP protein extraction solution (iNtRON Biotechnology, Kyungki-Do, Korea) or by ProFEK protein fraction enrichment kit (ITSIBIO, Johnstown, PA, USA), and the protein concentration was determined using a BCA protein assay kit (Bio-Rad Laboratories, Hercules, CA, USA). Equal amounts of cell lysate were separated by 10% or 12% SDS–PAGE and transferred to a polyvinylidene membrane (HybondTM-P, Amershan, Piscataway, NJ, USA). The blots were probed with the anti-iNOS (BD transduction Lab., San Jose, CA, USA), anti-Lamin and anti-tubulin (GeneTex Inc., Irvine, CA, USA), anti-COX-2, anti-p65, anti-p38, anti-p-p38, anti-ERK, anti-p-ERK, anti-JNK, anti-p-JNK, anti-ATF-2 and anti-MSK1 (Cell Signaling, Beverly, MA, USA) and anti-Actin (Sigma-Aldrich) antibodies. Antibodies were diluted in TBS (pH 7.5) containing 0.05% (v/v) Tween 20 and 5% (w/v) dried milk. Blots were incubated with the appropriate horseradish peroxidase-conjugated secondary antibodies (Jackson ImmunoResearch, West Grove, PA, USA). Bound antibodies were visualized by electrochemical luminescence staining (Western Lighting Plus ECL; PerkinElmer, Wellesley, MA, USA) with autoradiography using FUJI Medical x-ray film (FUJI Corporation, Kofu, Yamanashi, Japan).

### Cell viability assay

Macrophage cells were seeded in 24-well plates with 6×10^5^ cells per well in 1 ml culture medium. The cells were pretreated with or without different concentrations of compounds for 30 min and co-treated with LPS (100 ng/ml) for 24 h. The cultured medium was removed and the remaining cells were treated with MTT (3-(4,5-dimethylthiazol-2-yl)-2,5-iphenyltetrazolium bromide; 2 mg/ml; Sigma) and incubated at 37°C for 1 h. The MTT solution was removed and dimethyl sulfoxide (DMSO; Merck, Darnstadt, Germany) was added to solubilize the formazan crystals in the cells. The DMSO solution was quantified at 595 nm wavelength using a spectrophotometer (BioTek Instruments, Inc.). Each experiment was repeated at least 3 times. The ID50 value was defined as the dose of drugs at which 50% cell death occurred after 24 h of treatment.

### Animal model

Female BALB/C mice were purchased from BioLasco Taiwan Co., Ltd (Taipei, Taiwan), housed in laminar flow cabinets under specific pathogen-free conditions, and provided with sterilized food and water. Animal experiments were performed in accordance with Guidebook of Council of Agriculture for the Care and Use of Laboratory, and were approved by Institutional Animal Care and Use Committee (IACUC) of Fu-Jen Catholic University. Mice were pretreated with/without FJU-C4 compound (1 mg/kg or 5 mg/kg solved in DMSO/PBS buffer) for 30–60 min and then stimulated with a lethal dose of LPS (15 mg/kg) in PBS buffer. The serum of mice in each group was collected at 6 and 24 hrs. All mice were sacrificed at 24 hrs and the serum samples were collected by centrifugation. The amount of TNFα and IL-1β in the serum was determined by the standard curve using ELISA kit. To evaluate the efficacy of FJU-C4 against sepsis, mice were divided into three groups, including (1) LPS (15 mg/kg)+ solvent control, (2) LPS+ 5 mg/kg FJU-C4 and (3) LPS+10 mg/kg, and the procedure was carried out as previously described. The treated mice were observed every 4 hours, and the survival rates were calculated for one week.

### Statistical Methods

Results were presented as means ± SD. Data were presented as means and their 95% confidence intervals for at least 3 experiments. Data between groups were compared using the t-test. A *p*-value of <0.05 was considered statistically significant.

## Results

### Pyridone derivatives suppress iNOS and COX2 production induced by lipopolysaccharide stimulation in murine macrophages

**Figure 2:**
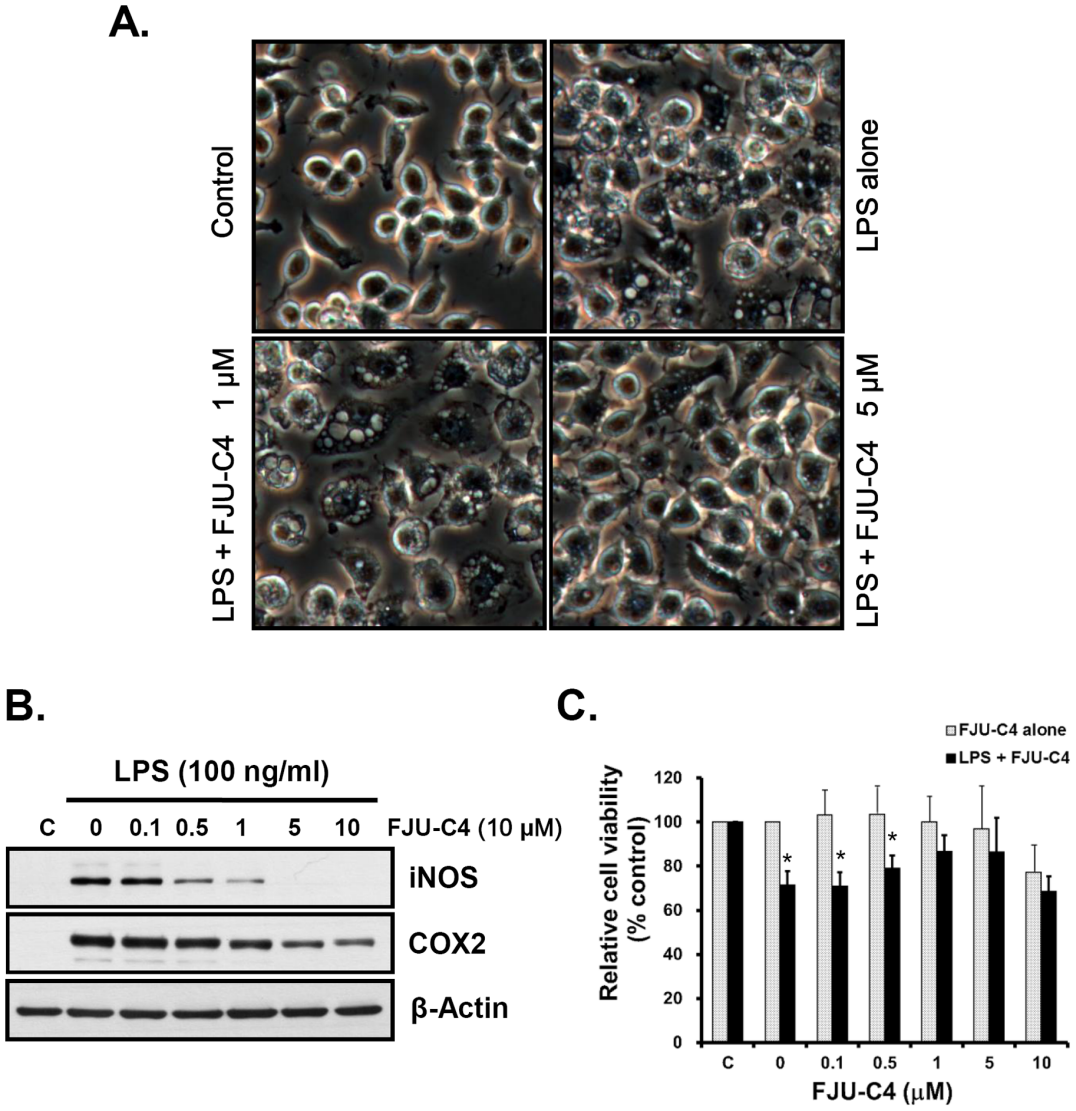
Suppressive effect of FJU-C4 compound on the activation of Raw264.7 murine macrophages. Raw264.7 macrophage cells were pretreated with various concentrations of FJU-C4 compound for 30 min and then stimulated by LPS (100 ng/ml) for 24 h. (A) The morphologically treated cells were observed by through a microscope (400×). (B) The production of iNOS and COX2 was analyzed by western blotting. (C) Cell viability of macrophage cells exposed to FJU-C4 alone, or with co-treated LPS and FJU-C4 was measured by MTT assay (*p<0.05 versus FJU-C4 alone). Data represent the mean ± SD of four independent experiments.

The synthesized compounds (C1–C7) were analyzed to determine whether they could inhibit the activation of macrophage cells induced by LPS stimulation. To prove this hypothesis, we pretreated Raw264.7 murine macrophage cells with 10 μM of various synthesized compounds (Fig. 1A) for 30 min and then stimulated the cells with 100 ng/ml LPS for 24 h. The treated cells were harvested and analyzed by western blotting, as shown in Fig. 1B. The results show that FJU-C4 compounds suppressed the production of iNOS (NOS2) and COX2 proteins dramatically. To further optimize the window dosage for the anti-inflammatory treatment of macrophage activation, we co-treated the cells with LPS (100 ng/ml) and various concentrations of FJU-C4 compound to monitor the inflammatory response for 24 h. As shown in Fig. 2A, the LPS-stimulated activation of Raw264.7 murine macrophage cells changed the cells morphologically to dendritic-like cells with multiple vacuoles in cytoplasm, whereas the untreated cells were round and small. The numbers of cells with activated morphology dramatically decreased when cells were dose-dependently pretreated with the FJU-C4 compound. We also monitored the production of iNOS and COX2 protein under parallel conditions by western blot analysis. The results were consistent with the findings on morphological alterations, wherein the production of iNOS and COX2 dose-dependently decreased as the dose of FJU-C4 compound increased. However, the inhibitory effect of the FJU-C4 compound was stronger on the expression of iNOS protein than on the production of COX2 protein (Fig. 2B). To elucidate whether the inhibitory effect on the production of iNOS and COX2 was caused by cell death, we measured cell viability by MTT assay under the treatment of novel FJU-C4 compounds with/without LPS stimulation. The results showed that the FJU-C4 compound exhibited low cell cytotoxicity in Raw264.7 macrophage cells below 5 μM. On the contrary, the FJU-C4 compound protected the Raw264.7 macrophage cells from LPS-induced apoptosis (Fig. 2C).

### 2-Pyridone derivatives suppress the expression of pro-cytokine genes in murine macrophages

**Figure 3:**
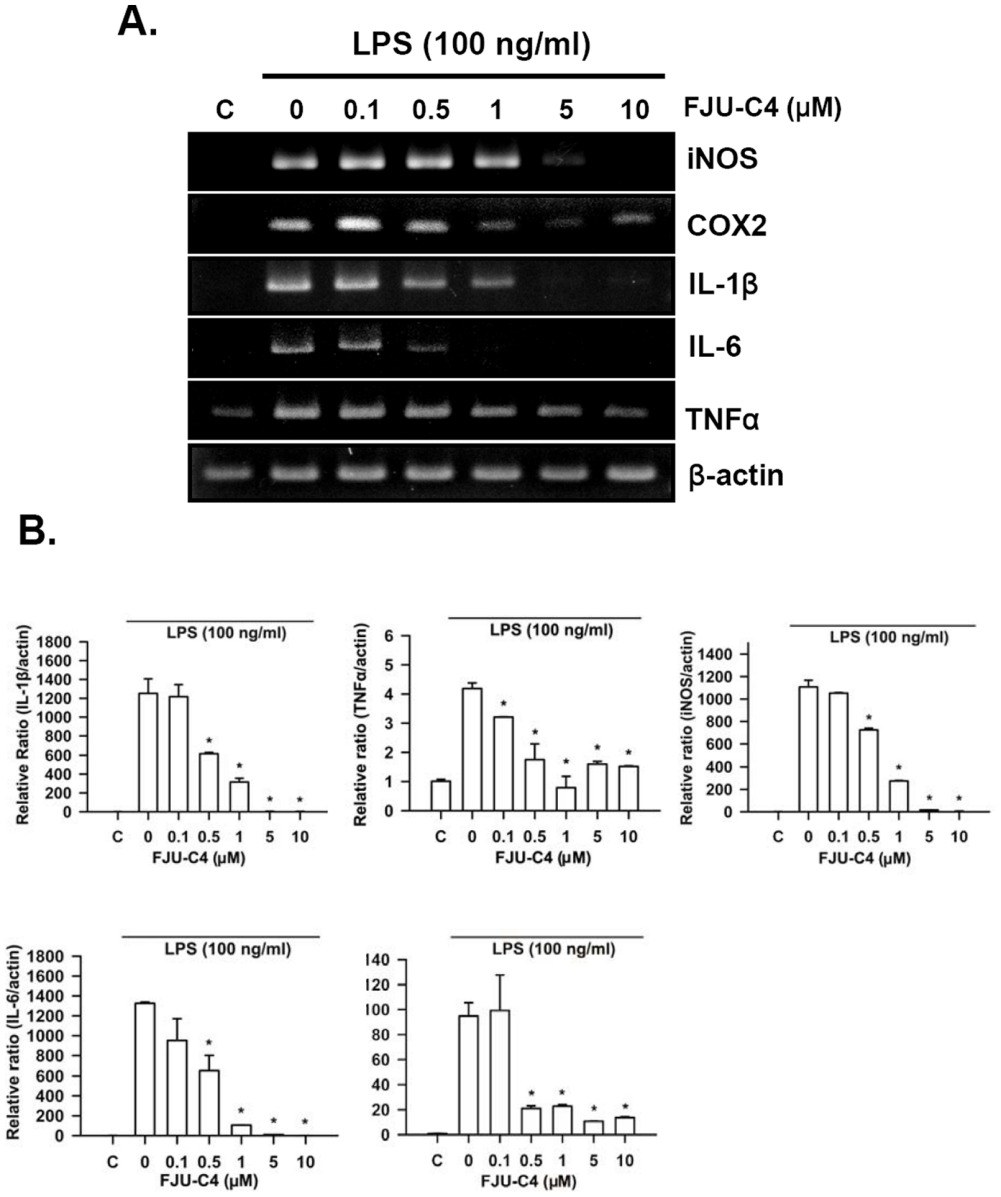
Inhibitory effect of FJU-C4 compound on the transcription level of pro-inflammatory cytokines. (A) Raw264.7 macrophage cells were pretreated with FJU-C4 compound from 0 to 10 μM for 30 min at the indicated dose and then stimulated by LPS (100 ng/ml) for 20 h. The mRNA of iNOS, COX2, TNFα and IL-1β and IL-6 was detected by RT-PCR with specific primers, as shown in Table 2. The amplified DNA fragment was analyzed by 1–1.2% agarose gel and visualized by ethidium bromide staining. (B) Quantitative real-time RT-PCR was performed with the specific primers, as listed in Table 2. The expression of β-actin was used as an internal control for RNA quantity (*p<0.05 versus the LPS group).

To evaluate the potential mechanism of these compounds in inhibiting inflammation, we measured the expression of procytokine genes, including TNFα, IL-1β, and IL-6 in LPS-stimulated Raw264.7 macrophage cells under the treatment with LPS and various concentrations of FJU-C4 compounds, using RT-PCR analysis. The results showed that the mRNA expression of iNOS and COX2 genes was dose-dependently downregulated when treated with an increased concentration of FJU-C4 compound (Fig. 3A). This result is consistent with the findings on protein levels detected by western blot analysis (Fig. 2B). The mRMA expression of precytokine genes, including TNFα, IL-1β, and IL-6, were also dramatically suppressed by the FJU-C4 compound treatment in a dose dependent manner at 20 h. To further confirm the findings, we designed the specific primers of these genes (Table 2) for SYBR Green real-time RT-PCR to quantify the ratio changes among treatment groups. The results showed that FJU-C4 dramatically inhibited the expression of iNOS, IL-1β and IL-6 genes at a dose of 1 μM and almost completely inhibited the expression of these genes when the dose was over 5 μM. Furthermore, it also suppressed the expression of COX2 and TNFα genes at a dose of 1 μM but did not cause further inhibition when the dose was increased, even when the dose was over 10 μM (Fig. 3B).

**Figure 4:**
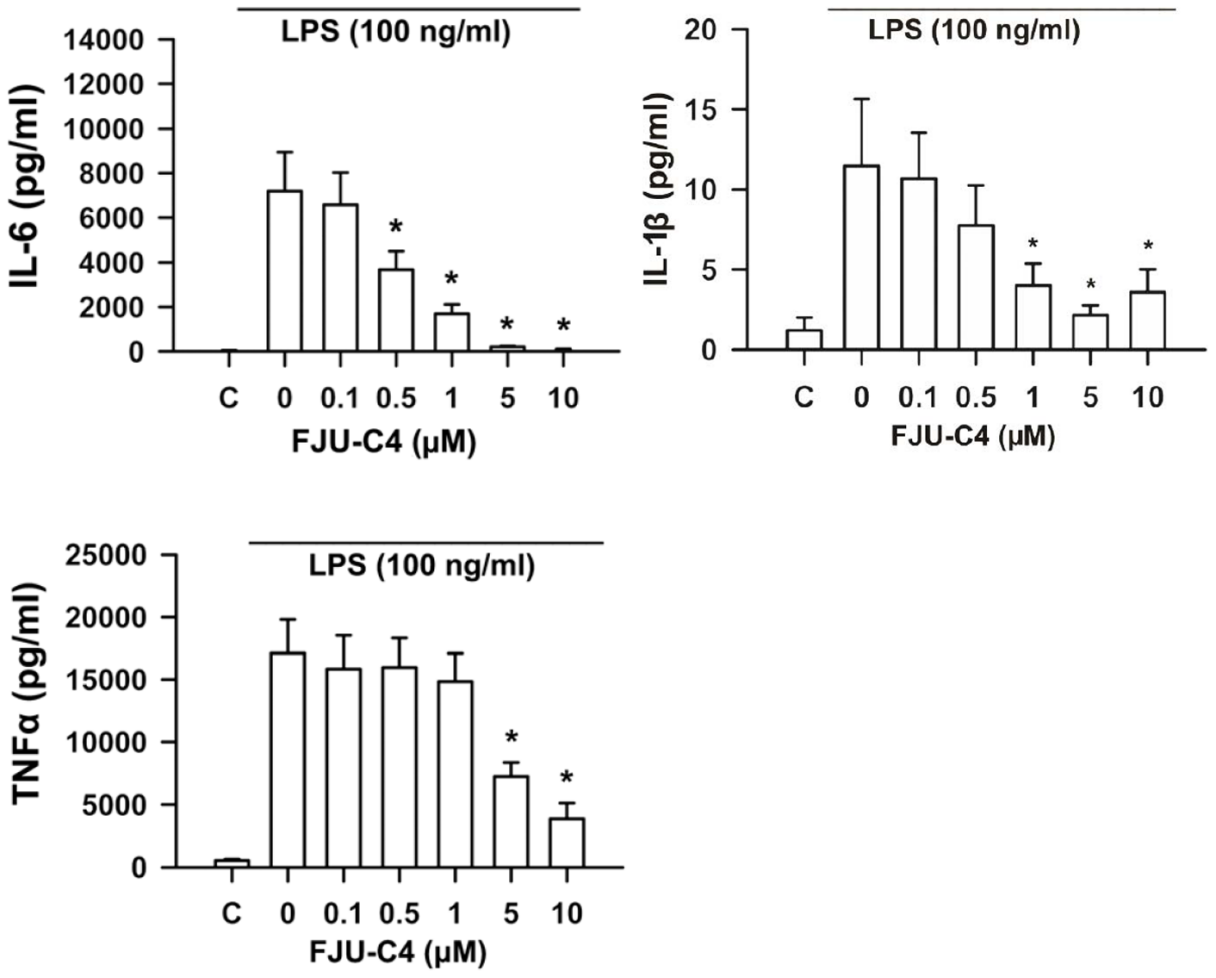
Amount of released mature pro-inflammatory cytokines was suppressed by FJU-C4 compound. Raw264.7 macrophage cells were pretreated with FJU-C4 compound at the indicated dose from 0 to 10 μM for 30 min and then stimulated by LPS (100 ng/ml) for 20 h. The amount of mature TNFα and IL-1β and IL-6 released in the culture medium was measured by ELISA. Values are means of 3 experiments in duplicate (*p<0.05 versus the LPS group).

We also measured the production of mature procytokines secreted in culture medium by ELISA assay. The results also showed that the FJU-C4 compound dose-dependently inhibited the secretion of IL-6, IL-1β, and TNFα (Fig. 4). The suppressive effect of FJU-C4 compound on the release of mature procytokines was consistent with the effect on the transcription level.

### FJU-C4 compound blocks inflammation by inhibiting p-38 and NF-κB signaling pathways

**Figure 5:**
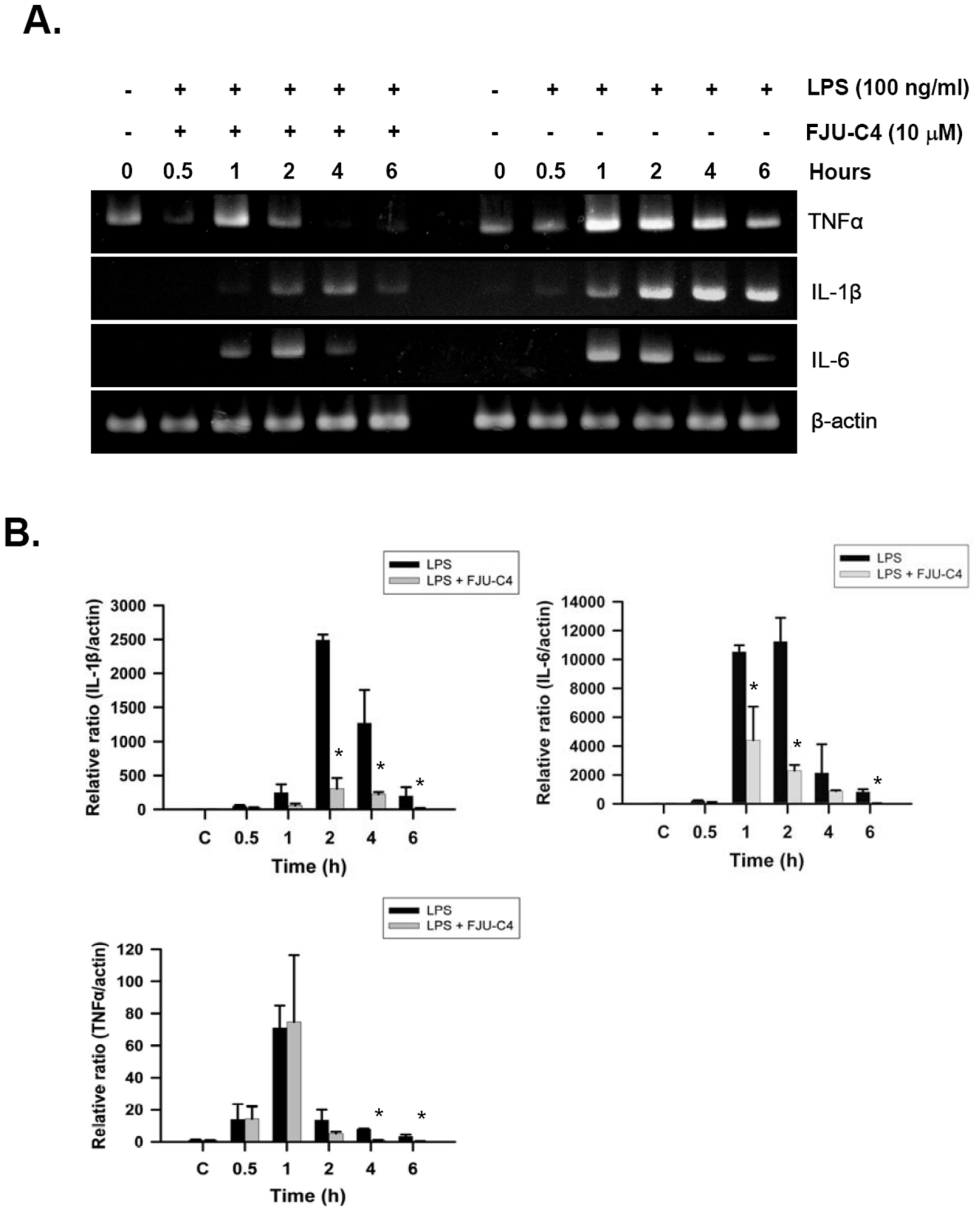
Effect of FJU-C4 on LPS-induced TNFα and IL-1β and IL-6 expression in Raw264.7 murine macrophages. Raw264.7 macrophage cells were pretreated with FJU-C4 (10 μM) compound for 30 min and then stimulated by LPS (100 ng/ml) for 0.5–6 h. (A) The mRNA levels of TNFα and IL-1β and IL-6 in the whole cell lysate were detected by RT-PCR. (B) Quantitative real-time RT-PCR was performed with the specific primers. The expression of β-actin served as an internal control for RNA quantity. Values are the means of the 3 experiments in duplicate (*p<0.05 versus the LPS group).

**Figure 6:**
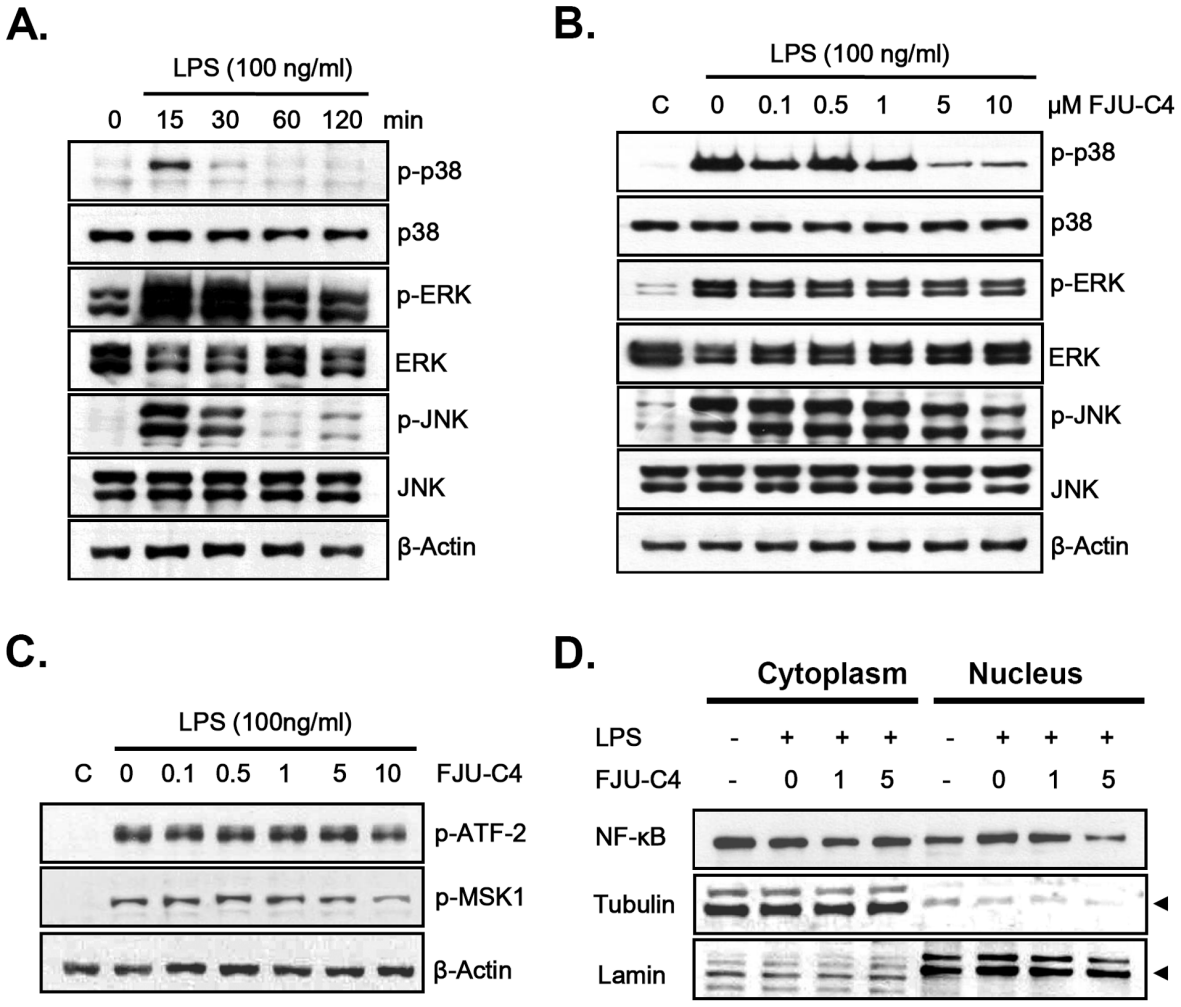
Effect of FJU-C4 on LPS-induced MAPKs phosphorylation and NF-κB activation in Raw264.7 murine macrophage culture. (A) Raw264.7 macrophage cells were pretreated with FJU-C4 (10 μM) compound for 30 min and then stimulated by LPS (100 ng/ml) for 15–60 min. (B) Raw264.7 macrophage cells were pretreated with various doses of FJU-C4 compound for 30 min and then stimulated by LPS (100 ng/ml) for 15 min. The activation of ERK, p38, and JNK was analyzed by western blotting, with specific anti- p-ERK, p-p38, and p-JNK antibodies, respectively. (C) The p38 downstream targets including ATF-2 and MSK1 were also measured by using specific antibodies. (D) Raw264.7 macrophage cells were pretreated with various doses of FJU-C4 compound for 30 min and then stimulated by LPS (1 μg/ml) for 60 min. The cytoplasm and nucleus fraction were collected by a protein fraction enrichment kit. The location of NF-κB was analyzed by western blotting with specific anti-NF-κB (p65) antibody. The tubulin and lamin proteins were detected as internal control for cytoplasm and nucleus fraction, respectively.

To identify the potential inhibitory mechanism of FJU-C4 on LPS-induced signaling pathways, we monitored changes in procytokine expression for up to 6 h. As shown in Fig. 5A, TNFα and IL-6 expressed the highest level of mRNA at the first hour and then decreased time dependently, but IL-1β gradually expressed the highest level of mRNA at 4 h when Raw264.7 macrophages were exposed to LPS stimulation. Conversely, co-treatment with FJU-C4 generally suppressed the expression level of these 3 procytokines. Fold change was measured and calculated by real-time PCR (Fig. 5B). Because previous studies have shown that the activation of mitogen-activated protein kinases (MAPKs) and NF-κB involved the induction of procytokines stimulated by LPS in murine macrophage cells, we further investigated the effect of FJU-C4 on the activation of MAPKs and NF-κB in LPS-stimulated Raw264.7 macrophage cells. The results showed that the phosphorylation of ERK, p38, and JNK peaked after 15 min and then gradually decreased (Fig. 6A). Therefore, we further measured the effect of FJU-C4 on the activation of MAPKs at 15 min. Co-treatment with FJU-C4 (5 μM and 10 μM) inhibited LPS-induced phosphorylation of p38-MAP kinase, but it produced slight effects on the activation of ERK and JNK (Fig. 6B). The activation of p38 downstream targets including ATF-2 and MSK1 were slightly mediated by FJU-C4 compound at high dose (Fig. 6C). In addition, FJU-C4 compound also blocked the LPS-induced NF-κB translocation from cytoplasm to nucleus in a dose-dependent manner (Fig. 6D).

### FJU-C4 compound inhibits pro-inflammatory cytokine production and prevents LPS-induced mouse death

**Figure 7:**
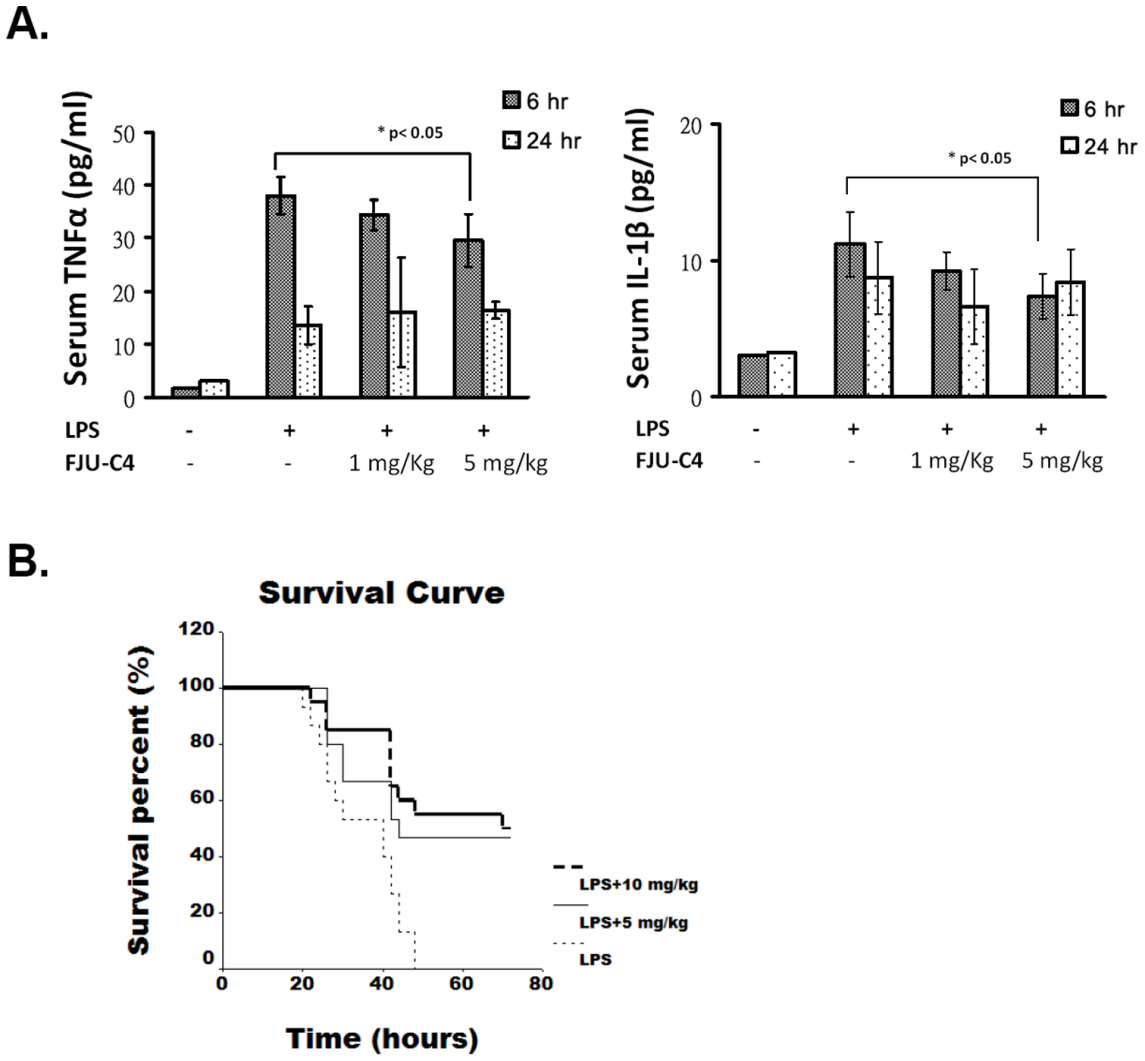
FJU-C4 compound inhibited pro-inflammatory cytokine secretion and prevented LPS-induced mouse death. Mice were pretreated with different doses of FJU-C4 compound as indicated and then stimulated by LPS (15 mg/kg) in PBS buffer (A) The serum of the mice (n = 3 for each group) was collected and measured for TNFα and IL-1β at 6 and 24 hours, respectively, by using ELISA kit with a standard protein curve. (B) LPS-treated mice with/without FJU-C4 compound treatment (as indicated) were observed for 72 hours and their survival rates were recorded. Mice in the control group were administered LPS alone with solvent (1% DMAO in PBS) (n = 15); one group was administered LPS plus 5 mg/kg FJU-C4 (n = 15), and the other group was administered LPS plus 10 mg/kg FJU-C4 (n = 20). The animal model data were pooled for three independent experiments. *p< 0.05 versus the LPS group.

To evaluate the suppressive effect of FJU-C4 compound on systemic inflammation induced by endotoxemia, mice were administrated a high dose of LPS (15 mg/kg) by intraperitoneal injection and the secretion of systemic cytokines in the blood was monitored by the ELISA method. The results showed that LPS injection by IP in mice enhanced systemic TNFα and IL-1β secretion as early as 6 hours after injection, after which such secretion declined at 24 hours. Pre-treatment with the FJU-C4 compound attenuated the systemic inflammation induced by LPS stimulation by decreasing the secretion of pro-inflammatory cytokines (Fig. 7A). In addition, FJU-C4 compound prevented LPS-induced mouse death when the dose was over 5 mg/kg, and the survival rate was almost near 50% either at 5 mg/kg or 10 mg/kg at 72 hours. The control mice were dead within 48 hours (Fig. 7B).

## Discussion

Cultured murine macrophage cells, Raw264.7, stimulated with LPS/INF-gama have become a common model of acute inflammation [17]. We have established the platform to screen the potential agents that can suppress the expression of iNOS and COX2 in activated macrophages for the treatment of acute inflammation, including acute inflammatory diseases. In this study, we demonstrated that the FJU-C4 compound is a novel, synthesized 2-pyridone compound derived from sulfur-substituted quinolizidines, however its structure is different from that of quinolizidine, with a cleavage on one ring. Some studies have demonstrated the biological function of related 2-pyridone compounds. Pirfenidone, a pyridone-related compound, has been reported to inhibit the production of TNF in vitro and in vivo and completely inhibit septic shock and subsequent mortality [18]. Other pyridone derivatives, such as 5-ethyl-1-phenyl-2-(1H) pyridine and fluorofenidone (AKF-PD), have been proved to protect mice from lethal endotoxemia induced by LPS stimulation in a mice model of septic shock by reducing the release of proinflammatory cytokines such as IL-1, IL-6 and TNFα [19], [20]. Other pryidone derivatives may be promising lead compounds with anti-inflammatory activities for the treatment of human diseases with dysregulated or excessive inflammatory responses [21], [22]. However, few studies have demonstrated the underlying anti-inflammatory response mechanisms of these related 2-pyridone compounds. In this study, we reported a new 2-pyridone related compound (FJU-C4) that produces an anti-inflammatory response to the activation of LPS-induced murine macrophage cells by inhibiting p38-MAP kinase as well as NF-κB, and prevents mice from LPS-induced death with systemic inflammation. The results provide a new spectrum of synthesized compounds in which FJU-C4 can be a good lead compound for developing potent anti-inflammation agents to reduce mortality caused by endotoxin-stimulated systemic inflammatory response syndrome, and for preventing subsequent multiorgan dysfunctions (MODs). However, the animal model of LPS-induced systemic inflammation by LPS is unable to interpret the human sepsis because there is no infectious pathogens were involved. Thus, the cecal ligation and puncture (CLP) mouse model, inducing polymicrobial bacterial peritonitis, should be further used to evaluate the potential clinical application of FJU-C4 on the treatment of human sepsis.

Nitric oxide (NO) generated by inducible nitric oxide synthase (iNOS) in activated macrophages is a crucial molecule for mediating biological functions, such as vasodilatation, neurotransmission, and inflammation, which are involved in inflammatory and autoimmune-mediated tissue destruction [23], [24], [25]. Cyclooxygenase-2 (COX-2) catalyzes the rate-limited step leading to the formation of prostaglandins (PGs), which are produced in high levels in inflamed tissues. The overexpression of COX-2 results from crosstalk among several mediators of inflammation, and it occurs through transcriptional activation. Targeting COX-2 expression may be a promising strategy for treating autoimmune diseases and cancer, as well as for avoiding severe side effects of COX-2 enzymatic inhibition [26], [27], [28]. Several studies have demonstrated that LPS mediates iNOS and COX-2 expression through MAPK and NF-κB signaling pathways [29], [30], [31], [32]. The activation of p38 MAPK signaling pathways is associated with the phagocytosis of bacteria by macrophages and is involved in human diseases such as sepsis [33] and chronic obstructive pulmonary disease (COPD) [34]. Inhibition of p38MAPK activity by either knock-down of gene or chemical inhibitors can inhibit the LPS-induced activation of macrophages and cause mice to become resistant to LPS-induced shock [35], [36]. Therefore, FJU-C4 potentially contributes to anti-sepsis through the inhibition of p-38 pathway activated by endotoxin stimulation. The animal model proved the hypothesis, but FJU-C4 was unable to fully protect all mice from LPS-induced death. However, the higher efficacy of modified 2-pyridone compounds in anti-inflammation and anti-sepsis must be further analyzed and their underlying mechanism should be further elucidated.

Our previous study demonstrated that a new aza-Diels-Alder reaction of thio-substituted 3-sulfolenes with p-toluenesulfonyl isocyanate (PTSI) can synthesize several sulfur-substituted piperidine derivatives [11]. Based on this method, a series of quinolizinones and pyridones were synthesized in this study to evaluate their biological function in anti-inflammatory responses. Although compound C6 also contains 2-pyridone, it exhibits no effects on anti-inflammatory responses. This result indicated that the function group “6-Allyl-” may contribute a significant motif in the FJU-C4 compound, compared to the structure of C6-compounds for the inhibition of LPS-induced activation of macrophage cells. Previous studies demonstrated that LPS stimulates macrophage activation through the Toll-like receptor 4 (TLR4)-p38MAPK signaling pathway [37]. This means that the FJU-C4 compound containing active moiety may interact with a crucial mediator to abolish signaling transduction from the TLR4 receptor complex to p38MAPK. Downstream signaling of the TLR4 receptor complex in response to LPS is regulated by adapter proteins, including MyD88, MAL, TRIF, and TRAM. Therefore, the specific target protein interacted with the FJU-C4 compound, and the more potent compound derived from the FJU-C4 structure should be further identified and developed.
